# Genotypic Findings in Noonan and Non-Noonan RASopathies and Patient Eligibility for Growth Hormone Treatment

**DOI:** 10.3390/jcm12155003

**Published:** 2023-07-29

**Authors:** Atilano Carcavilla, Ana Cambra, José L. Santomé, Verónica Seidel, Jaime Cruz, Milagros Alonso, Jesús Pozo, Irene Valenzuela, Encarna Guillén-Navarro, Fernando Santos-Simarro, Isabel González-Casado, Amparo Rodríguez, Constancio Medrano, Juan Pedro López-Siguero, Begoña Ezquieta

**Affiliations:** 1 Pediatric Endocrinology Department, Hospital Universitario La Paz, 28046 Madrid, Spain; 2 Multidisciplinary Unit for RASopathies, Hospital Universitario La Paz, 28046 Madrid, Spain; 3 Molecular Diagnostics Laboratory, Department of Laboratory Medicine, Hospital General Universitario Gregorio Marañón, 28007 Madrid, Spain; 4 Gregorio Marañon Health Research Institute (IiSGM), 28009 Madrid, Spain; 5 Clinical Genetics Unit, Pediatrics Department, Hospital General Universitario Gregorio Marañón, 28007 Madrid, Spain; 6 Pediatrics Department, Hospital Universitario Doce de Octubre, 28041 Madrid, Spain; 7 Pediatrics Department, Hospital Ramón y Cajal, 28034 Madrid, Spain; 8 Pediatric Endocrinology Department, Hospital Universitario Niño Jesús, 28009 Madrid, Spain; 9 Genetics Department, Hospital Universitario Vall D’Hebrón, 08035 Barcelona, Spain; 10 Genetics Department, Hospital Virgen de la Arrixaca, 30120 Murcia, Spain; 11 Institute of Medical & Molecular Genetics, Hospital Universitario la Paz, 28046 Madrid, Spain; 12 Pediatric Endocrinology, Pediatrics Department, Hospital General Universitario Gregorio Marañón, 28007 Madrid, Spain; 13 Pediatric Cardiology Department, Hospital General Universitario Gregorio Marañón, 28007 Madrid, Spain; 14 Pediatric Endocrinology Department, Hospital Regional Universitario de Málaga, 29010 Málaga, Spain

**Keywords:** short stature, growth, growth hormone, RASopathies, Noonan syndrome, genetic, *PTPN11*, Ras/MAPK pathway

## Abstract

Molecular study has become an invaluable tool in the field of RASopathies. Treatment with recombinant human growth hormone is approved in Noonan syndrome but not in the other RASopathies. The aim of this study was to learn about the molecular base of a large cohort of patients with RASopathies, with particular emphasis on patients with pathogenic variants in genes other than *PTPN11*, and its potential impact on rGH treatment indication. We reviewed the clinical diagnosis and molecular findings in 451 patients with a genetically confirmed RASopathy. *HRAS* alterations were detected in only 2 out of 19 patients referred with a Costello syndrome suspicion, whereas pathogenic variants in *RAF1* and *SHOC2* were detected in 3 and 2, respectively. In 22 patients referred with a generic suspicion of RASopathy, including cardiofaciocutaneous syndrome, pathogenic alterations in classic Noonan syndrome genes (*PTPN11*, *SOS1*, *RAF1*, *LZTR1,* and *RIT1*) were found in 7 patients and pathogenic variants in genes associated with other RASopathies (*HRAS*, *SHOC2,* and *PPPCB1)* in 4. The correct nosological classification of patients with RASopathies is critical to decide whether they are candidates for treatment with rhGH. Our data illustrate the complexity of differential diagnosis in RASopathies, as well as the importance of genetic testing to guide the diagnostic orientation in these patients.

## 1. Introduction

Noonan syndrome (NS) is a multisystem genetic disorder characterized by manifestations such as short stature, distinctive craniofacial dysmorphism, congenital heart disease, ectodermal and skeletal anomalies, mild and variable developmental delay, and predisposition to myeloproliferative disorders. Its frequency is unknown, although its incidence is estimated to be between 1:1000 and 1:2500 live births [1]. *PTPN11* was the first causative gene identified in NS in 2001 and is considered to be responsible for the condition in approximately 50% of patients [2]. *PTPN11* codes for SHP2, a protein–tyrosine–phosphatase involved in the RAS/mitogen-activated protein kinase (MAPK) pathway, an intracellular signaling cascade that is essential for cell proliferation, differentiation, and survival. Several overlapping genetic disorders, such as Costello syndrome, cardiofaciocutaneous syndrome (CFCS), NS with multiple lentigines (NSML, formerly known as LEOPARD syndrome), NS-like with loose anagen hair (NSLAH, also known as Mazzanti syndrome), and neurofibromatosis type 1 are caused by alterations in genes encoding components of the RAS/MAPK pathway. As a result, these entities have been collectively named “RASopathies” [3]. Short stature is a cardinal feature of NS, and its severity varies according to the genotype [4]. It is also a common feature in other rasopathies, particularly in CFCS, CS, and NSLAH [5].

Genetic studies in the last two decades have identified more than 20 different genes involved in RASopathies [6], and efforts have been made to provide formal evidence-based classifications for the association of a gene with a given disease [7]. Molecular testing has become an invaluable tool for clinicians attempting to confirm or reorient a clinical diagnosis. However, while there is extensive information about the phenotypic profile of patients with NS due to alterations in common genes (e.g., *PTPN11*), less is known about the clinical particularities of patients with NS due to pathogenic variants in other less common genes [6]. In addition, nosological classifications of affected patients are essential when considering treatment since recombinant human growth hormone (rhGH) is approved in NS but not in the other RASopathies [4,8].

This study presents genotypic findings from a large cohort of patients with a clinical suspicion of NS or other RASopathies in order to improve our understanding of the molecular basis of the disease and its potential impact on the decision to start rhGH. 

## 2. Materials and Methods

We reviewed the clinical diagnosis and molecular findings of 451 patients with clinical suspicion of Noonan or other, related non-Noonan RASopathies with a positive finding in molecular testing (i.e., pathogenic or likely pathogenic variants, as defined by the ClinVar and/or NSeuronet gene databases) in RASopathy-related OMIM genes after gene sequencing (stratified monogenic Sanger) or massive panel sequencing. Patients were referred for molecular testing between January 2005 and July 2022. Fifty-seven patients were found to carry variants of unknown significance (VUS, uncertain or conflicting interpretation in ClinVar, revised 21 September 2022) and were not included in this study. The analyses were requested by specialists involved in the care of these patients (i.e., pediatric endocrinologists, clinical geneticists, and pediatric cardiologists) from 65 hospitals in 14 Spanish regions. Blood or DNA samples were sent to our center from participating hospitals by the attending clinicians, who had previously obtained the patients’ informed consent. An initial clinical evaluation based on the preanalytical questionnaire designed by Ezquieta et al. [9] was used for case selection. In 391 patients, stratified Sanger sequencing, according to our usual practice at that moment [9], including the recurrent regions of *BRAF*, *CBL*, *HRAS*, *KRAS*, *MAP2K1*, *NRAS*, *PTPN11*, *RAF1*, *RIT1*, *SHOC2*, and *SOS1,* allowed us to characterize the pathogenic alteration. Sequencing was performed bidirectionally using fluorescent dideoxynucleotides and the ABI Prism W3100 device (Instrumental Line Sequencing, Genomics Unit, Hospital General Universitario Gregorio Marañón). The results were analyzed using SeqScape 2.5. Sanger sequencing was also performed to confirm variants and for family studies (57 family members carrying the pathogenic alteration). Most patients with a well-recognized pathogenic variant did not undergo further NGS analysis, with some exceptions, namely, those with greater than expected clinical severity and, importantly, all the patients with a VUS finding in the Sanger analyses. Some of the VUS detected in the monogenic analyses of *PTPN11* are now recognized as pathogenic or likely-pathogenic variants in ClinVar (c.1471 C>A p.Pro491Thr; c.155 C>T p.Thr52Ile; c.217-218 AC>CT p.Thr73Leu; c.781 C>T p.Leu261Phe; c.328 G>A p.Glu110Lys; c.774 G>T p.Glu258Asp) or have been already reported by other authors in ClinVar and were considered pathogenic based on their de novo appearance and extremely low frequency in the general population in gnomAD (c.28A>G p.Asn10Asp) following the criteria in the ACMG guidelines [10].

NGS sequencing was performed in 238 patients using a customized in-house panel for RASopathies with 25 genes, including *A2ML1*, *ARID2*, *BRAF, CBL, HRAS*, *KAT6B*, *KRAS*, *LZTR1*, *MAP2K1*, *MAP2K2*, *MAP3K8*, *NF1*, *PIK3R1*, *PTPN11*, *PPP1CB1*, *RAF1*, *RASA1*, *RASA2*, *RIT1*, *RRAS*, *SHOC2*, *SOS1*, *SOS2*, *SPRED1*, and *SPRY1* (the underlined genes are Noonan and RASopathy genes recognized as causative by OMIM). *MRAS*, *RRAS2*, *MAPK1*, and *SPRED2* were incorporated in the upgraded designs of capture probe pools, as they were considered Noonan genes by OMIM. The remaining genes in the panel were Noonan candidate genes or genes found to be mutated in diseases with Noonan-related clinical signs. Nextera Flex libraries (Illumina, San Diego, CA, USA) and capture-enrichment with IDT probes (Integrated DNA Technologies, Coralville, IA, USA) were used. The MiSeq platform from Illumina with a NanoSeq High Output Reagent Kit (300-cycles) Alignment and variant calling were applied using MiSeq Reporter v. 2.6.2.3. (Illumina), followed by annotation of VCF output files using BaseSpace Variant Interpreter (Illumina) and Mutation Taster 2021 [11,12]. Genetic variants were filtered based on population frequency and pathogenic prediction criteria using 6 publicly available databases (ClinVar, ensemble, NS Euronet, HGMD, gnomAD, and TopMed). Variants not previously described were evaluated in silico using Polyphen-2 [13], Mutation Taster [11], and SIFT [14] version 4.0.3 (J. Craig Venter Institute; http://sift.jcvi.org). When available, parental DNA analyses determined whether inheritance was de novo or the recessive form in *LZTR1*.

## 3. Results

Table 1 summarizes the number of patients with each RASopathy and the genes involved in Noonan and non-Noonan RASopathies. Unexpected genotypic findings based on the initial clinical suspicion are presented in Table 2. Detailed genotypic findings, initial clinical suspicion, and final diagnosis of the whole cohort are presented in Supplemental Table S1.

Nineteen patients were referred with suspected Costello syndrome (n = 8) or with generic suspicion of RASopathy, including Costello syndrome (n = 11). Alterations in *HRAS* were detected in only two of these patients. The remaining genes involved were *RAF1* (three patients, all with Costello syndrome suspicion), *RIT1* (two patients), *BRAF* (one patient)*, MAP2K1* (three patients), *MAP2K2* (two patients), *PTPN11* (two patients), *SOS1* (one patient), and *SHOC2* (three patients, two of them with Costello syndrome suspicion).

Twenty-two patients were referred with suspected CFCS (n = 7) or with generic suspicion of RASopathy, including CFCS (n = 15). Eleven patients carried variants in genes commonly associated with CFCS, as follows, *BRAF* (seven patients), *MAP2K1* (one patient), *MAP2K2* (two patients), and *KRAS* (one patient). However, pathogenic alterations in classic NS genes were found in seven of these patients: *PTPN11* (two), *SOS1* (two), *RAF1* (one), *LZTR1* (one), and *RIT1* (one). Furthermore, genetic analysis revealed variants in genes associated with other RASopathies in four patients: *HRAS* (one patient), *SHOC2* (two patients), and *PPPCB1* (one patient).

An additional group of 18 patients were finally diagnosed with CFCS after molecular testing and clinical re-evaluation. Thirteen of these patients had an initial clinical suspicion of NS, and 5 were suspected of having other RASopathies.

## 4. Discussion

This report focused on patients whose genotypes involved non-*PTPN11* genes, irrespective of whether an NS or other non-Noonan RASopathy had been suspected, with emphasis on data relating to the suitability of rhGH treatment.

After initial approval for treatment with rhGH in NS by the United States Food and Drug Administration in 2007, other countries, such as Brazil, Israel, Japan, and South Korea, began to treat NS patients. More recently, rhGH was approved in the European Union under a mutual recognition procedure based on an initial authorization granted by Denmark in 2020. The conditions for initiating treatment vary according to local health authorities, although they often depend on genetic confirmation and expressly exclude non-Noonan RASopathies, despite their strong clinical overlap [15].

Findings in NSLAH overlap considerably with those of NS and other RASopathies (eg, Costello syndrome and CFCS syndrome) [16]. In NSLAH, the most distinctive signs are easily pluckable, sparse, thin, slow-growing hair (loose anagen hair); hyperpigmented skin; a hypernasal voice; and a higher frequency of short stature due to growth hormone deficiency [17]. Nevertheless, some of these manifestations are age-dependent or difficult to assess, and it is often challenging for the clinician to distinguish between NSLAH and NS, and even between NSLAH and Costello syndrome. Affected patients also have a distinctive genotypic profile, with alterations in *SHOC2* [18] or in *PPP1CB* [19].

In our series, Costello syndrome or CFCS syndrome were initially suspected in 6 patients with NSLAH (3 with the recurrent *SHOC2* alteration and 2 with the *SHOC2* alteration and another with a pathogenic *PPP1CB* alteration, respectively; see Table 2 and Supplemental Table S1). Therefore, rhGH would not have been considered indicated in these patients. While information about the efficacy and safety of rhGH in CFCS and Costello syndrome is scarce, some authors reported a good response to rhGH in NSLAH [17,20]. Furthermore, NSLAH may be associated with a lower risk of malignancies than the other RASopathies [21,22]. However, as a non-Noonan RASopathy, NSLAH is not an indication for rhGH in some European countries, despite most of the available data on the efficacy and safety profile of rhGH coming from observational studies on patients without genetic confirmations [4]. Three other patients with initial suspicion of CS received a new diagnosis of NS after identifying a variant in *RAF1* (see Table 2). Due to this diagnostic reorientation, these patients may be candidates for GH treatment.

Furthermore, 7 patients out of 22 with a clinical suspicion of RASopathy, including CFCS, had a genotype compatible with NS, which probably led to a reconsideration of their clinical label. If they had remained under the clinical diagnosis of CFCS, they would not have been considered candidates for rhGH. Conversely, several patients with an initial clinical diagnosis of NS were found to carry *BRAF* alterations. This molecular finding could have prompted a reassessment of the phenotype and in some cases could lead to the alternative diagnosis of CFCS since *BRAF,* and also *KRAS*, are considered an NS and CFCS gene by OMIM (NS7/CFCS1 and NS3/CFCS2, respectively) [8,23]. As these patients meet the clinical criteria for NS, they should not be excluded from treatment with rhGH.

Genotypes may help to guide surveillance if a gene—or even a specific variant—strongly associated with a particular clinical course is detected. Of note, one of our patients with a clinical suspicion of CFCS had a mutation in *HRAS*. Careful re-evaluation of the patient led to the diagnosis of Costello syndrome, and a cancer screening protocol was initiated, along with other changes in the patient’s follow-up. However, regardless of genotype, some clinical manifestations should be taken into account when a treatment with rhGH is being considered. Intellectual disability (IQ < 70) is uncommon in NS patients [1], whereas it is much more frequent in other rasopathies such as CFCS or CS [24,25]. As treatment with rhGH in patients with intellectual disability raises ethical questions [26], this manifestation should be taken into consideration in this setting.

One of the main strengths of our study lies in the large sample size analyzed and the variety of centers involved in the study. This has allowed us to obtain relevant information when assessing the adequacy of growth hormone treatment in patients with rasopathies. Among its limitations, updated clinical information after genotypic diagnosis was not always available, which makes it difficult to establish a definitive diagnosis in certain patients.

The correct nosological classifications of patients with RASopathies are critical when deciding whether they are candidates for treatment with rhGH. Our data illustrate the complexity of the differential diagnosis within RASopathies, as well as the importance of the genetic study for guiding the diagnosis and management in these patients.

## Figures and Tables

**Table 1:** Patients with pathogenic or likely pathogenic variants in Noonan-related genes. Alterations in genes other than *PTPN11* were detected in 135 patients. Only unrelated patients are considered in this study; 57 additional patients were family members in 43 families, involving *PTPN11* (n = 34), *SOS1* (n = 6), *LZTR1* (n = 2), and *NF1* (n = 1).

	RASopathy	n = 451
	Noonan syndrome	357
	*PTPN11*, *SOS1*, *BRAF* ^a^, *RAF1*, *RIT1*, *KRAS* ^b^, *LZTR1 (dom and rec)*, and *NRAS*	
Non NS RASopathies	Noonan syndrome with multiple lentigines	47
	*PTPN11, RAF1,* and *BRAF* ^b^	
	Cardiofaciocutaneous syndrome	27
	*BRAF* ^a^, *KRAS* ^b^, *MAP2K1*, and *MAP2K2*	
	Costello syndrome	3
	*HRAS* ^c^	
	Noonan-like syndrome with loose anagen hair	6
	*SHOC* ^d^ and *PPP1CB*	
	Neurofibromatosis—Noonan syndrome	11
	*NF1*	
No pathogenic alterations were detected in the remaining OMIM RASopathy genes: *CBL*, *MRAS*, *RRAS2*, *MAPK1*, *SPRED1*, and *SPRED2* ^e^.		

^a^. See Table 2, which shows 9 patients with an initial clinical suspicion of NS and a final diagnosis of CFCS and 1 patient with NSML phenotype. ^b^. Five patients with *KRAS* pathogenic variants in this series, one with CFC (see Table 2), another with juvenile myelomonocytic leukemia, and the remaining 3 with an NS phenotype. ^c^. See Table 2. ^d^. Five patients with a different previous diagnosis: 3 NS, 1 NSML, and 1 Legius syndrome. ^e^. Patients with variants of as yet unknown clinical significance (uncertain or conflicting interpretations in ClinVar or NSEuronet) were not included: 39 VUS in OMIM Noonan and RASopathy-causing genes (*LZTR1* n = 8, *SOS1* n = 10, *NF1* n = 9, *RIT1* n = 2, and *SHOC2* n = 4; and *BRAF*, *RAF1*, *MAP2K1*, *MAP2K2*, *SPRED1*, and *SPRED2*, n = 1 each) and 18 VUS in candidate or Noonan phenotype–related genes (*A2ML1, ARID2, RASA2, KAT6B, MAP3K8, SPRY1,* and *PIK3R1*). No pathogenic alterations were identified in the latter group.

**Table 2:** Unexpected genotypic findings based on the initial clinical suspicion.

	Genes in Which Pathogenic Alterations Were Detected in Each Group of Patients with an “Unexpected Genotype”											
Clinical Suspicion (Number of Patients)	NS OMIM Genes					NS and Non-NS OMIM Genes		Non NS RASopathy-Genes				
	*PTPN11*	*SOS1*	*RAF1*	*RIT1*	*LZTR1*	*BRAF*	*KRAS*	*HRAS*	*MAP2K1*	*MAP2K2*	*SHOC2*	*PPP1CB*
Costello syndrome (n = 8)			3			1		2			2	
Costello syndrome and other RASopathies (n = 11)	2	1		2					3	2	1	
CFC (n = 7)						6		1				
CFC and other RASopathies (n = 15)	2	2	1	1	1	1	1		1	2	2	1
NS suspected, diagnosis of CFC after genotyping (n = 13)						9			4			
Other suspected RASopathies, diagnosis of CFC after genotyping (n = 5)						2	1		2			

## Data Availability

Not applicable.

## Supplementary Materials

The following supporting information can be downloaded at: https://www.mdpi.com/article/10.3390/jcm12155003/s1, Table S1: Full list of patients with genotypic findings and initial and final diagnosis.
