# Supplementary material for: Genotypic Findings in Noonan and Non-Noonan RASopathies and Patient Eligibility for Growth Hormone Treatment
Source: J Clin Med. 2023 Jul 29;12(15):5003. doi: 10.3390/jcm12155003 (PMC10420167; doi:10.3390/jcm12155003)
Supplement: Supplementary file 1 [file jcm-12-05003-s001.zip › jcm-2490499-supplementary.pdf]

Supplemental Table S1: Full list of patients with genotypic findings and initial and final diagnosis.

Patient	Genotype	*GENE*	Diagnostic suspicion	Final diagnosis[a]
1	c.922 A>G (p.Asn308Asp)	*PTPN11*	NS	NS
2	c.770 C>T (p.Ser257Leu)	*RAF1*	NS	NS
3	c.1403 C>T (p.Thr468Met)	*PTPN11*	NS	NS
4	c.1403 C>T (p.Thr468Met)	*PTPN11*	NS	NSML
5	c.170C>G (p.Ala57Gly)	*RIT1*	NS	NS
6	c.184 T>G (p.Tyr62Asp)	*PTPN11*	NS	NS
7	c.1510 A>G (p.Met504Val)	*PTPN11*	NS	NS
8	c.806 T>C (p.Met269Thr)	*SOS1*	NS	NS
9	c.854 T>C (p.Phe285Ser)	*PTPN11*	NS	NS
10	c.797 C>A (p.Thr266Lys)	*SOS1*	NS	NS
11	c.179 G>C (p.Gly60Ala)	*PTPN11*	NS	NS
12	c.1403 C>T (p.Thr468Met)	*PTPN11*	NFNS	NSML
13	c.218 C>T (p.Thr73Ile)	*PTPN11*	NS	NS
14	c.1656 G>T (p-Arg552Ser)	*SOS1*	NS	NS
15	c.806 T>C (p.Met269Thr)	*SOS1*	NS	NS
16	c.922 A>G (p.Asn308Asp)	*PTPN11*	NS	NS
17	c.214 G>T (p.Ala72Ser)	*PTPN11*	NS	NS
18	c.922 A>G (p.Asn308Asp)	*PTPN11*	NS	NS
19	c.922 A>G (p.Asn308Asp)	*PTPN11*	NS	NS
20	c.782 C>A (p.Pro124Gln)	*MAP2K1*	NS	CFCS
21	c.1654 A>G (p.Arg552Gly)	*SOS1*	NS	NS
22	c.922 A>G (p.Asn308Asp)	*PTPN11*	NS	NS
23	c.853 T>C (p.Phe285Leu)	*PTPN11*	NS	NS
24	c.417 G>C (p.Glu139Asp)	*PTPN11*	NS	NS
25	c.922 A>G (p.Asn308Asp)	*PTPN11*	NS	NS
26	c.854 T>G (p.Phe285Cys)	*PTPN11*	NS	NS
27	c.782 C>A (p.Pro261His)	*RAF1*	NS	NS
28	c.181 G>A (p.Asp61Asn)	*PTPN11*	NS	NS
29	c.922 A>G (p.Asn308Asp)	*PTPN11*	NS	NS
30	c.1510 A>G (p.Met504Val)	*PTPN11*	NS	NS
31	c.417 G>C (p.Glu139Asp)	*PTPN11*	NS	NS
32	c.1510 A>G (p.Met504Val)	*PTPN11*	NS	NS
33	c.1403 C>T (p.Thr468Met)	*PTPN11*	NS	NSML
34	c.767 A>G (p.Gln256Arg)	*PTPN11*	NS	NS
35	c.170C>G p.Ala57Gly	*RIT1*	NS	NS
36	c.836 A>G (p.Tyr279Cys)	*PTPN11*	NSML	NSML
37	c.236 A>G (p.Gln79Arg)	*PTPN11*	NS	NS
38	c.922 A>G (p.Asn308Asp)	*PTPN11*	NS	NS
39	c.182 A>G (p.Asp61Gly)	*PTPN11*	NS	NS
40	c.199G>A (p.Asp67Asn)	*MAP2K1*	NS	CFCS
41	c.124 A>G (p.Thr42Ala)	*PTPN11*	NS	NS
42	c.172 A>C (p.Asn58His)	*PTPN11*	NS	NS
43	c.1403 C>T (p.Thr468Met)	*PTPN11*	NS	NSML

44	c.124 A>G (p.Thr42Ala)	*PTPN11*	NS	NS
45	c.770C>T (p.Ser257Leu)	*RAF1*	NS	NS
46	c.4A>G (p.Ser2Gly)	*SHOC2*	CFCS among others	NSLAH
47	c.797 C>A (p.Thr266Lys)	*SOS1*	NS	NS
48	c.155 C>T (p.Thr52Ile)	*PTPN11*	NS	NS
49	c.923 A>G (p.Asn308Ser)	*PTPN11*	NS	NS
50	c.923 A>G (p.Asn308Ser)	*PTPN11*	NS	NS
51	c.172 A>G (p.Asn58Asp)	*PTPN11*	NS	NS
52	c.1471 C>T (p.Pro491Ser)	*PTPN11*	NS	NS
53	c.854 T>C (p.Phe285Ser)	*PTPN11*	NS	NS
54	c.182 A>G (p.Asp61Gly)	*PTPN11*	NS	NS
55	c.104G>C (p.Ser35Thr)	*RIT1*	NS	NS
56	c.1528 C>G (p.Gln510Glu)	*PTPN11*	NS	NSML
57	c.181 G>A (p.Asp61Asn)	*PTPN11*	NS	NS
58	c.236 A>G (p.Gln79Arg)	*PTPN11*	NS	NS
59	c.284G>C (p.Gly95Ala)	*RIT1*	NS	NS
60	c.797 C>A (p.Thr266Lys)	*SOS1*	NS	NS
61	c.844 A>G (p.Ile282Val)	*PTPN11*	NS	NS
62	c.922 A>G (p.Asn308Asp)	*PTPN11*	NS	NS
63	c.246T>A (p.Phe82Leu)	*RIT1*	NS	NS
64	c.188 A>G (p.Tyr63Cys)	*PTPN11*	NS	NS
65	c.1510 A>G (p.Met504Val)	*PTPN11*	NS	NS
66	c.104G>C (p.Ser35Thr)	*RIT1*	NS	NS
67	c.1655 G>C (p.Arg552Thr)	*SOS1*	NSML	NS
68	c.417 G>C) (p.Glu139Asp)	*PTPN11*	NS	NS
69	c.806T>G (p.Met269Arg)	*SOS1*	CFCS&CS among others	NS
70	c.214 G>T (p.Ala72Ser)	*PTPN11*	NS	NS
71	c.5716del (p.Leu1906SerfsTer19)	*NF1*	NFNS	NFNS
72	c.389A>G (p.Tyr130Cys)	*MAP2K1*	CFCS&CS among others	CFCS
73	c.770C>T (p.Ser257Leu)	*RAF1*	CS	NS
74	c.1403 C>T (p.Thr468Met)	*PTPN11*	NFNS	NSML
75	c.1471C>A (p.Pro491Thr)	*PTPN11*	NS	NS
76	c.214 G>T (p.Ala72Ser)	*PTPN11*	NS	NS
77	c.1642 A>C (p.Ser548Arg)	*SOS1*	NS	NS
78	c.2197A>T (p.Ile733Phe)	*SOS1*	CFCS among others	NS
79	c.184 T>G (p.Tyr62Asp)	*PTPN11*	NS	NS
80	c.922 A>G (p.Asn308Asp)	*PTPN11*	NS	NS
81	c.922 A>G (p.Asn308Asp)	*PTPN11*	NS	NS
82	c.922 A>G (p.Asn308Asp)	*PTPN11*	NS	NS
83	c.215 C>G (p.Ala72Gly)	*PTPN11*	NS	NS
84	c.922 A>G (p.Asn308Asp)	*PTPN11*	NS	NS
85	c.172 A>G (p.Asn58Asp)	*PTPN11*	NS	NS
86	c.922 A>G (p.Asn308Asp)	*PTPN11*	NSML	NS
87	c.1529 A>G (p.Gln510Arg)	*PTPN11*	NS	NSML
88	c.1510 A>G (p.Met504Val)	*PTPN11*	NS	NS

89	c.182 A>G (p.Asp61Gly)	*PTPN11*	NS	NS
90	c.853 T>C (p.Phe285Leu)	*PTPN11*	NS	NS
91	c.923 A>G (p.Asn308Ser)	*PTPN11*	NS	NS
92	c.923 A>G (p.Asn308Ser)	*PTPN11*	NS	NS
93	c.179 G>C (p.Gly60Ala)	*PTPN11*	NS	NS
94	c.214 G>T (p.Ala72Ser)	*PTPN11*	NS	NS
95	c.1529 A>C (p.Gln510Pro)	*PTPN11*	NS	NS
96	c.2536G>A (p.Glu846Lys)	*SOS1*	NS	NS
97	c.922 A>G (p.Asn308Asp)	*PTPN11*	NS	NS
98	c.217-218 AC>CT (p.Thr73Leu)	*PTPN11*	NS	NS
99	c.236 A>G (p.Gln79Arg)	*PTPN11*	NS	NS
100	c.124 A>G (p.Thr42Ala)	*PTPN11*	NS	NS
101	c.215 C>G (p.Ala72Gly)	*PTPN11*	NS	NS
102	c.1403 C>T (p.Thr468Met)	*PTPN11*	NSML	NSML
103	c.922 A>G (p.Asn308Asp)	*PTPN11*	NS	NS
104	c.923 A>G (p.Asn308Ser)	*PTPN11*	NS	NS
105	c.417 G>C (p.Glu139Asp)	*PTPN11*	NS	NS
106	c.1504 T>G (p.Ser502Ala)	*PTPN11*	NS	NS
107	c.922 A>G (p.Asn308Asp)	*PTPN11*	NS	NS
108	c.922 A>G (p.Asn308Asp)	*PTPN11*	NS	NS
109	c.214 G>T (p.Ala72Ser)	*PTPN11*	NS	NS
110	c.1403 C>T (p.Thr468Met)	*PTPN11*	NSML	NSML
111	c.768_771dupCAA	*PTPN11*	NS	NS
112	c.5425C>T (p.Arg1809Cys)	*NF1*	NSML	NFNS
113	c.922 A>G (p.Asn308Asp)	*PTPN11*	NS	NS
114	c.770A>G (p.Gln257Arg)	*BRAF*	CFCS	CFCS
115	c.1507 G>A (p.Gly503Arg)	*PTPN11*	NS	NS
116	c.853 T>C (p.Phe285Leu)	*PTPN11*	NS	NS
117	c.925 G>T (p.Asp309Tyr)	*SOS1*	NS	NS
118	c.922 A>G (p.Asn308Asp)	*PTPN11*	NS	NS
119	c.781 C>T (p.Leu261Phe)	*PTPN11*	NS	NS
120	c.2183 A>T (p.Lys728Ile)	*SOS1*	NS	NS
121	[c.188 A>G (p.Tyr63Cys); c.931A>G (p.Met311Val)]	*PTPN11*	NS	NS
122	c.1403 C>T (p.Thr468Met)	*PTPN11*	NS	NSML
123	c.770C>T (p.Ser257Leu)	*RAF1*	NFNS	NSML
124	c.770C>T (p.Ser257Leu)	*RAF1*	NS	NS
125	c.923 A>G (p.Asn308Ser)	*PTPN11*	NS	NS
126	c.1510 A>G (p.Met504Val)	*PTPN11*	NS	NS
127	c.922 A>G (p.Asn308Asp)	*PTPN11*	NS	NS
128	c.1330_1332delATG	*SOS1*	NS	NS
129	c.1471 C>T (p.Pro491Ser)	*PTPN11*	NS	NS
130	c.1654 A>T (p.Arg552Trp)	*SOS1*	NS	NS
131	c.417 G>C (p.Glu139Asp)	*PTPN11*	NS	NS
132	c.844 A>G (p.Ile282Val)	*PTPN11*	NS	NS
133	c.218 C>T (p.Thr73Ile)	*PTPN11*	NS	NS
134	c.1432 G>A (p.Glu478Lys)	*RAF1*	NS	NS
135	c.922 A>G (p.Asn308Asp)	*PTPN11*	NS	NS

136	c.188 A>G (p.Tyr63Cys)	*PTPN11*	NS	NS
137	c.214 G>T (p.Ala72Ser)	*PTPN11*	NS	NS
138	c.768_771dupCAA	*PTPN11*	NS	NS
139	c.199G>A (p.Asp67Asn)	*MAP2K1*	NS	CFCS
140	c.768_771dupCAA	*PTPN11*	NS	NS
141	c.253 T>C (p.Trp85Arg)	*SOS1*	NS	NS
142	c.1501G>A (p.Glu501Lys)	*BRAF*	NS	CFCS
143	c.182 A>G (p.Asp61Gly)	*PTPN11*	NS	NS
144	c.922 A>G (p.Asn308Asp)	*PTPN11*	NS	NS
145	c.1510 A>G (p.Met504Val)	*PTPN11*	NS	NS
146	c.1501G>A (p.Glu501Lys)	*BRAF*	CFCS	CFCS
147	c.215 C>G (p.Ala72Gly)	*PTPN11*	NS	NS
148	c.922 A>G (p.Asn308Asp)	*PTPN11*	NS	NS
149	c.1510 A>G (p.Met504Val)	*PTPN11*	NS	NS
150	c.417 G>C (p.Glu139Asp)	*PTPN11*	NS	NS
151	c.923 A>G (p.Asn308Ser)	*PTPN11*	NS	NS
152	c.836 A>G (p.Tyr279Cys)	*PTPN11*	NSML	NSML
153	c.236 A>G (p.Gln79Arg)	*PTPN11*	NS	NS
154	c.922 A>G (p.Asn308Asp)	*PTPN11*	NS	NS
155	c.1741A>G (p.Asn581Asp)	*BRAF*	CFCS	CFCS
156	c.1654 A>G (p.Arg552Gly)	*SOS1*	NS	NS
157	c.317 A>C (p.Asp106Ala)	*PTPN11*	NS	NS
158	c.1654 A>G (p.Arg552Gly)	*SOS1*	NS	NS
159	c.188 A>G (p.Tyr63Cys)	*PTPN11*	NS	NS
160	c.1381 G>T (p.Ala461Ser)	*PTPN11*	NSML	NSML
161	c.244T>G (p.Phe82Val)	*RIT1*	CS among others	NS
162	c.922 A>G (p.Asn308Asp)	*PTPN11*	NS	NS
163	c.802 G>T (p.Gly268Cys)	*PTPN11*	NS	NS
164	c.922 A>G (p.Asn308Asp)	*PTPN11*	NS	NS
165	c.174 C>G (p.Asn58Lys)	*PTPN11*	NS	NS
166	c.182 A>G (p.Asp61Gly)	*PTPN11*	NS	NS
167	c.199G>A (p.Asp67Asn)	*MAP2K1*	CS among others	CFCS
168	c.221C>G (p.Ala74Gly)	*RIT1*	NS	NS
169	c.236 A>G (p.Gln79Arg)	*PTPN11*	NS	NS
170	c.776 C>T (p.Ser259Phe)	*RAF1*	NSML	NSML
171	c.923 A>G (p.Asn308Ser)	*PTPN11*	NS	NS
172	c.853 T>C (p.Phe285Leu)	*PTPN11*	NS	NS
173	c.178 G>A (p.Gly60Ser)	*PTPN11*	NS	NS
174	c.172 A>G (p.Asn58Asp)	*PTPN11*	NS	NS
175	c.922 A>G (p.Asn308Asp)	*PTPN11*	NS	NS
176	c.188 A>G (p.Tyr63Cys)	*PTPN11*	NS	NS
177	c.1510 A>G (p.Met504Val)	*PTPN11*	NS	NS
178	c.181 G>A (p.Asp61Asn)	*PTPN11*	NS	NS
179	c.317 A>C (p.Asp106Ala)	*PTPN11*	NS	NS
180	c.188 A>G (p.Tyr63Cys)	*PTPN11*	NS	NS
181	c.836 A>G (p.Tyr279Cys)	*PTPN11*	NS	NSML
182	c.922 A>G (p.Asn308Asp)	*PTPN11*	NS	NS
183	c.922 A>G (p.Asn308Asp)	*PTPN11*	NS	NS

184	c.836 A>G (p.Tyr279Cys)	*PTPN11*	NSML	NSML
185	c.188 A>G (p.Tyr63Cys)	*PTPN11*	NS	NS
186	c.188 A>G (p.Tyr63Cys)	*PTPN11*	NS	NS
187	c.1510 A>G (p.Met504Val)	*PTPN11*	NS	NS
188	c.781 C>A (p.Pro261Thr)	*RAF1*	NS	NS
189	c.124 A>G (p.Thr42Ala)	*PTPN11*	NS	NS
190	c.1294T>A (p.Trp432Arg)	*SOS1*	NS	NS
191	c.1381 G>A (p.Ala461Thr)	*PTPN11*	NS	NS
192	c.1510 A>G (p.Met504Val)	*PTPN11*	NS	NS
193	c.179 G>C (p.Gly60Ala)	*PTPN11*	NS	NS
194	c.188 A>G (p.Tyr63Cys)	*PTPN11*	NS	NS
195	c.1505 C>T) (p.Ser502Leu)	*PTPN11*	NS	NS
196	c.1294T>C (p.Trp432Arg)	*SOS1*	NS	NS
197	c.395G>A (p.Gly132Asp)	*MAP2K2*	CFCS&CS among others	CFCS
198	c.3827G>A (p.Arg1267Gln)	*NF1*	NS	NFNS
199	c.1654 A>G (p.Arg552Gly)	*SOS1*	NS	NS
200	c.922 A>G (p.Asn308Asp)	*PTPN11*	NS	NS
201	c.854 T>G (p.Val14Ile)	*KRAS*	NS	NS
202	c.328 G>A (p.Glu110Lys)	*PTPN11*	NS	NS
203	c.922 A>G (p.Asn308Asp)	*PTPN11*	NS	NS
204	c.417 G>C (p.Glu139Asp)	*PTPN11*	NS	NS
205	c.188 A>G (p.Tyr63Cys)	*PTPN11*	NS	NS
206	c.922 A>G (p.Asn308Asp)	*PTPN11*	NSML	NS
207	c.236 A>G (p.Gln79Arg)	*PTPN11*	NS	NS
208	c.770A>G (p.Gln257Arg)	*BRAF*	NSML	NSML
209	c.922 A>G (p.Asn308Asp)	*PTPN11*	NS	NS
210	c.922 A>G (p.Asn308Asp)	*PTPN11*	NS	NS
211	c.922 A>G (p.Asn308Asp)	*PTPN11*	NS	NS
212	c.1403 C>T (p.Thr468Met)	*PTPN11*	MSML	NSML
213	c.317 A>C (p.Asp106Ala)	*PTPN11*	NS	NS
214	c.922 A>G (p.Asn308Asp)	*PTPN11*	NS	NS
215	c.922 A>G (p.Asn308Asp)	*PTPN11*	NS	NS
216	c.922 A>G (p.Asn308Asp)	*PTPN11*	NS	NS
217	c.770A>G (p.Gln257Arg)	*BRAF*	NS	CFCS
218	c.1310 T>C) (p.Ile437Thr)	*SOS1*	NS	NS
219	c.770C>T (p.Ser257Leu)	*RAF1*	CS	NS
220	c.736G>C (p.Ala246Pro)	*BRAF*	NS	CFCS
221	c.922 A>G (p.Asn308Asp)	*PTPN11*	NS	NS
222	c.922 A>G (p.Asn308Asp)	*PTPN11*	NS	NS
223	c.922 A>G (p.Asn308Asp)	*PTPN11*	NS	NS
224	c.922 A>G (p.Asn308Asp)	*PTPN11*	NS	NS
225	c.922 A>G (p.Asn308Asp)	*PTPN11*	NS	NS
226	c.922 A>G (p.Asn308Asp)	*PTPN11*	NS	NS
227	c.4A>G (p.Ser2Gly)	*SHOC2*	CFCS&CS among others	NSLAH
228	c.836 A>G (p.Tyr279Cys)	*PTPN11*	NS	NSML
229	c.417 G>C (p.Glu139Asp)	*PTPN11*	NS	NS

230	c.922 A>G (p.Asn308Asp)	*PTPN11*	NS	NS
231	c.4A>G (p.Ser2Gly)	*SHOC2*	CS	NSLAH
232	c.236 A>G (p.Gln79Arg)	*PTPN11*	NS	NS
233	c.844 A>G (p.Ile282Val)	*PTPN11*	NS	NS
234	c.417 G>C (p.Glu139Asp)	*PTPN11*	NS	NS
235	c.922 A>G (p.Asn308Asp)	*PTPN11*	NS	NS
236	c.1310 T>C (p.Ile437Thr)	*SOS1*	NS	NS
237	c.922 A>G (p.Asn308Asp)	*PTPN11*	NS	NS
238	c.188 A>G (p.Tyr63Cys)	*PTPN11*	NS	NS
239	c.922 A>G (p.Asn308Asp)	*PTPN11*	NS	NS
240	c.236 A>G (p.Gln79Arg)	*PTPN11*	NS	NS
241	c.1471 C>T (p.Pro491Ser)	*PTPN11*	NS	NS
242	c.1455 G>T (p.Leu485Phe)	*BRAF*	CFCS among others	CFCS
243	c.1510 A>G (p.Met504Val)	*PTPN11*	NS	NS
244	c.922 A>G (p.Asn308Asp)	*PTPN11*	NS	NS
245	c.806T>G (p.Met269Arg)	*SOS1*	NS	NS
246	c.236 A>G (p.Gln79Arg)	*PTPN11*	NS	NS
247	c.1403 C>T (p.Thr468Met)	*PTPN11*	NS	NS
248	c.922 A>G (p.Asn308Asp)	*PTPN11*	NS	NS
249	c.1510 A>G (p.Met504Val)	*PTPN11*	NS	NS
250	c.742G>A (p.Gly248Arg)	*LZTR1*	NS	NS
251	c.1510 A>G (p.Met504Val)	*PTPN11*	NS	NS
252	c.246T>A (p.Phe82Leu)	*RIT1*	CFCS among others	NS
253	c.236 A>G (p.Gln79Arg)	*PTPN11*	NS	NS
254	c.34G>A (p.Gly12Ser)	*HRAS*	CS	Costello
255	c.2536 G>A (p.Glu846Lys)	*SOS1*	NS	NS
256	c.736G>C (p.Ala246Pro)	*BRAF*	CFCS	CFCS
257	c.284G>C (p.Gly95Ala)	*RIT1*	NS	NS
258	c.922 A>G (p.Asn308Asp)	*PTPN11*	NS	NS
259	c.3916C>T(p.Arg1306Ter)	*NF1*	NFNS	NFNS
260	c.184 T>G (p.Tyr62Asp)	*PTPN11*	NS	NS
261	c.188 A>G (p.Tyr63Cys)	*PTPN11*	NS	NS
262	c.59A>C (p.Gln20Pro)	*NF1*	NFNS	NFNS
263	c.1654 A>G (p.Arg552Gly)	*SOS1*	NS	NS
264	[c.2074T>C (p.Phe692Leu)];[c.2074T>C (p.Phe692Leu)]	*LZTR1*	CFCS among others	NS
265	c.1510 A>G (p.Met504Val)	*PTPN11*	NS	NS
266	c.922 A>G (p.Asn308Asp)	*PTPN11*	NS	NS
267	c.770A>G (p.Gln257Arg)	*BRAF*	NS	CFCS
268	c.922 A>G (p.Asn308Asp)	*PTPN11*	NS	NS
269	c.922 A>G (p.Asn308Asp)	*PTPN11*	CFCS among others	NS
270	c.1403 C>T (p.Thr468Met)	*PTPN11*	NS	NSML
271	c.1381 G>A (p.Ala461Thr)	*PTPN11*	NS	NS
272	c.922 A>G (p.Asn308Asp)	*PTPN11*	NSML	NS
273	c.170C>G (p.Ala57Gly)	*RIT1*	CS among others	NS
274	c.1403 C>T (p.Thr468Met)	*PTPN11*	NSML	NSML
275	c.922 A>G (p.Asn308Asp)	*PTPN11*	NS	NS

276	c.182 A>G (p.Asp61Gly)	*PTPN11*	NS	NS
277	c.782 C>T (p.Pro261Leu)	*RAF1*	NS	NS
278	c.836 A>G (p.Tyr279Cys)	*PTPN11*	NSML	NSML
279	c.317 A>C (p.Asp106Ala)	*PTPN11*	NS	NS
280	c.922 A>G (p.Asn308Asp)	*PTPN11*	NS	NS
281	c.4A>G (p.Ser2Gly)	*SHOC2*	CS	NSLAH
282	c.184 T>G (p.Tyr62Asp)	*PTPN11*	NS	NS
283	c.770C>T (p.Ser257Leu)	*RAF1*	CFCS among others	NS
284	c.1528 C>G (p.Gln510Glu)	*PTPN11*	NS	NS
285	c.1403 C>T (p.Thr468Met)	*PTPN11*	NS	NSML
286	c.174 C>G (p.Asn58Lys)	*PTPN11*	NS	NS
287	c.797 C>A (p.Thr266Lys)	*SOS1*	NS	NS
288	c.922 A>G (p.Asn308Asp)	*PTPN11*	NS	NS
289	c.389A>G (p.Tyr130Cys)	*MAP2K1*	NS	CFCS
290	c.1507 G>A (p.Gly503Arg)	*PTPN11*	NS	NSML
291	c.214 G>T (p.Ala72Ser)	*PTPN11*	NS	NS
292	c.181 G>A (p.Asp61Asn)	*PTPN11*	NS	NS
293	c.181 G>A (p.Asp61Asn)	*PTPN11*	NS	NS
294	c.246T>A (p.Phe82Leu)	*RIT1*	NS	NS
295	c.1507 G>A (p.Gly503Arg)	*PTPN11*	NS	NS
296	c.1492 C>T (p.Arg498Trp)	*PTPN11*	NSML	NSML
297	c.922 A>G (p.Asn308Asp)	*PTPN11*	NS	NS
298	c.188 A>G (p.Tyr63Cys)	*PTPN11*	NS	NS
299	c.1529 A>C (p.Gln510Pro)	*PTPN11*	NSML	NSML
300	c.836 A>G (p.Tyr279Cys)	*PTPN11*	NSML	NSML
301	c.188 A>G (p.Tyr63Cys)	*PTPN11*	NS	NS
302	c.922 A>G (p.Asn308Asp)	*PTPN11*	NS	NS
303	c.923 A>G (p.Asn308Ser)	*PTPN11*	NS	NS
304	c.1391 G>C (p.Gly464Ala)	*PTPN11*	NS	NSML
305	c.179 G>C (p.Gly60Ala)	*PTPN11*	NS	NS
306	c.922 A>G (p.Asn308Asp)	*PTPN11*	NS	NS
307	c.721A>C (p.Thr241Pro)	*BRAF*	NS	CFCS
308	c.854 T>C (p.Phe285Ser)	*PTPN11*	NS	NS
309	c.925 G>T (p.Asp309Tyr)	*SOS1*	NS	NS
310	c.770A>G (p.Gln257Arg)	*BRAF*	NS	CFCS
311	c.1300 G>C (p.Gly434Arg)	*SOS1*	NS	NS
312	c.922 A>G (p.Asn308Asp)	*PTPN11*	NS	NS
313	c.836 A>G (p.Tyr279Cys)	*PTPN11*	NSML	NSML
314	c.215 C>G (p.Ala72Gly)	*PTPN11*	NS	NS
315	c.922 A>G (p.Asn308Asp)	*PTPN11*	CS among others	NS
316	c.178G>C (p.Gly60Arg)	*KRAS*	CFCS among others	CFCS
317	c.853 T>C (p.Phe285Leu)	*PTPN11*	NS	NS
318	c.181 G>A (p.Asp61Asn)	*PTPN11*	NS	NS
319	c.1510 A>G (p.Met504Val)	*PTPN11*	NS	NS
320	c.922 A>G (p.Asn308Asp)	*PTPN11*	NS	NS
321	c.236 A>G (p.Gln79Arg)	*PTPN11*	NS	NS
322	c.376A>G (p.Asn126Asp)	*MAP2K2*	CFCS&CS among others	CFCS

323	c.836 A>G (p.Tyr279Cys)	*PTPN11*	NSML	NSML
324	c.1510 A>G (p.Met504Val)	*PTPN11*	NS	NS
325	c.1654 A>G (p.Arg552Gly)	*SOS1*	NS	NS
326	c.417 G>C (p.Glu139Asp)	*PTPN11*	NS	NS
327	c.791+1G>T	*LZTR1*	NS	NS
328	c.205 G>C (p.Glu69Gln)	*PTPN11*	NS	NS
329	c.1837C>G (p.Leu613Val	*RAF1*	NS	NS
330	c.508A>G (p.Lys170Glu)	*SOS1*	NS	NS
331	c.922 A>G (p.Asn308Asp)	*PTPN11*	NS	NS
332	c.922 A>G (p.Asn308Asp)	*PTPN11*	NS	NS
333	c.4700C>A (p.Ser1567Ter)	*NF1*	Legius	NFNS
334	c.1528 C>G (p.Gln510Glu)	*PTPN11*	NS	NSML
335	c.215 C>G (p.Ala72Gly)	*PTPN11*	NS	NS
336	c.844 A>G (p.Ile282Val)	*PTPN11*	NS	NS
337	c.1510 A>G (p.Met504Val)	*PTPN11*	NS	NS
338	c.770C>T (p.Ser257Leu)	*RAF1*	CS	NS
339	c.179 G>C (p.Gly60Ala)	*PTPN11*	NS	NS
340	c.188 A>G (p.Tyr63Cys)	*PTPN11*	NS	NS
341	c.1403 C>T (p.Thr468Met)	*PTPN11*	NS	NSML
342	c.417 G>C (p.Glu139Asp)	*PTPN11*	NS	NS
343	c.188 A>G (p.Tyr63Cys)	*PTPN11*	NS	NS
344	c.251C>T (p.Ala84Val)	*RIT1*	NS	NS
345	c.1403 C>T (p.Thr468Met)	*PTPN11*	NS	NSML
346	c.1741A>G (p.Asn581Asp)	*BRAF*	CS	CFCS
347	c.172 A>G (p.Asn58Asp)	*PTPN11*	NS	NS
348	c.1528 C>G (p.Gln510Glu)	*PTPN11*	NS	NSML
349	c.218 C>T (p.Thr73Ile)	*PTPN11*	JMML	JMML
350	c.1654 A>G (p.Arg552Gly)	*SOS1*	NS	NS
351	c.188 A>G (p.Tyr63Cys)	*PTPN11*	NS	NS
352	c.1528 C>G (p.Gln510Glu)	*PTPN11*	NS	NSML
353	c.774 G>T (p.Glu258Asp)	*PTPN11*	NS	NS
354	c.922 A>G (p.Asn308Asp)	*PTPN11*	NS	NS
355	c.923 A>G (p.Asn308Ser)	*PTPN11*	NS	NS
356	c.922 A>G (p.Asn308Asp)	*PTPN11*	NS	NS
357	c.836 A>G (p.Tyr279Cys)	*PTPN11*	NS	NSML
358	c.844 A>G (p.Ile282Val)	*PTPN11*	NS	NS
359	c.806 T>C (p.Met269Thr)	*SOS1*	NS	NS
360	c.836 A>G (p.Tyr279Cys)	*PTPN11*	NSML	NSML
361	c.1403 C>T (p.Thr468Met)	*PTPN11*	NSML	NSML
362	c.1802A>C (p.Lys601Thr)	*BRAF*	CFCS	CFCS
363	c.124 A>G (p.Thr42Ala)	*PTPN11*	NS	NS
364	c.923 A>G (p.Asn308Ser)	*PTPN11*	NS	NS
365	c.389A>G (p.Tyr130Cys)	*MAP2K1*	CS among others	CFCS
366	c.922 A>G (p.Asn308Asp)	*PTPN11*	NS	NS
367	c.548A>T (p.Glu183Val)	*PPP1CB*	CFCS among others	NSLAH
368	c.922 A>G (p.Asn308Asp)	*PTPN11*	NS	NS
369	c.922 A>G (p.Asn308Asp)	*PTPN11*	NS	NS

370	c.64C>A (p.Gln22Lys)	*HRAS*	CFCS	Costello
371	c.1381 G>A (p.Ala461Thr)	*PTPN11*	CFCS&CS among others	NS
372	c.781C>T (p.Pro261Ser)	*RAF1*	NS	NS
373	c.417 G>C (p.Glu139Asp)	*PTPN11*	NS	NS
374	c.922 A>G (p.Asn308Asp)	*PTPN11*	NS	NS
375	c.1510 A>G (p.Met504Val)	*PTPN11*	NS	NS
376	c.923 A>G (p.Asn308Ser)	*PTPN11*	NS	NS
377	c.188 A>G (p.Tyr63Cys)	*PTPN11*	NS	NS
378	c.3330delT (p.Phe1110LeufsTer2)	*NF1*	NS	NFNS
379	c.417 G>C (p.Glu139Asp)	*PTPN11*	NS	NS
380	c.1510 A>G (p.Met504Val)	*PTPN11*	NS	NS
381	c.1084 C>T p.Arg362Ter)	*LZTR1*	NS	NS
382	c.1501G>A (p.Glu501Lys)	*BRAF*	CFCS	CFCS
383	c.922 A>G (p.Asn308Asp)	*PTPN11*	NS	NS
384	c.1403 C>T (p.Thr468Met)	*PTPN11*	NSML	NSML
385	c.172 A>C (p.Asn58His)	*PTPN11*	NS	NS
386	c.188 A>G (p.Tyr63Cys)	*PTPN11*	NS	NS
387	c.844 A>G (p.Ile282Val)	*PTPN11*	NS	NS
388	c.806 T>C (p.Met269Thr)	*SOS1*	NS	NS
389	c.922 A>G (p.Asn308Asp)	*PTPN11*	NS	NS
390	c.1507 G>A (p.Gly503Arg)	*PTPN11*	NS	NS
391	c.317 A>C (p.Asp106Ala)	*PTPN11*	NS	NS
392	c.242A>G (p.Glu81Gly)	*RIT1*	NS	NS
393	c.1301 G>A (p.Gly434Arg)	*SOS1*	NS	NS
394	c.922 A>G (p.Asn308Asp)	*PTPN11*	NS	NS
395	c.776C>G (p.Ser259Cys)	*RAF1*	NS	NS
396	c.174 C>A (p.Asn58Lys)	*PTPN11*	NS	NS
397	c.172 A>G (p.Asn58Asp)	*PTPN11*	NS	NS
398	c.214 G>T (p.Ala72Ser)	*PTPN11*	NS	NS
399	c.1318C>T (p.Arg440Ter)	*NF1*	NFNS	NFNS
400	c.1472C>A (p.Pro491His)	*PTPN11*	NS	NSML
401	c.40G>A (p.Val14Ile); c.7756G>T (p.Glu2586*)	*KRAS; NF1*	NFNS	NS & NF1
402	c.236 A>G (p.Gln79Arg)	*PTPN11*	NS	NS
403	c.2970_2972delAAT (p.Met992del)	*NF1*	NS	NFNS
404	c.7348C>T (p.Arg2450Ter)	*NF1*	NFNS	NFNS
405	c.4A>G (p.Ser2Gly)	*SHOC2*	NSLSH	NSLAH
406	c.35G>A (p.Gly12Asp) mosaicism	*KRAS*	JMML	JMML
407	c.1403 C>T (p.Thr468Met)	*PTPN11*	NSML	NSML
408	c.188 A>G (p.Tyr63Cys)	*PTPN11*	NSML	NS
409	c.417 G>C (p.Glu139Asp)	*PTPN11*	NS	NS
410	[c.2074T>C (p.Phe692Leu)]; [c.2070-2A>G]	*LZTR1*	NS	NS
411	c.1654 A>G (p.Arg552Gly)	*SOS1*	NS	NS
412	c.770C>T (p.Ser257Leu)	*RAF1*	NS	NS

413	c.1510 A>G (p.Met504Val)	*PTPN11*	NS	NS
414	c.170C>G (p.Ala57Gly)	*RIT1*	NS	NS
415	c.284G>C (p.Gly95Ala)	*RIT1*	NS	NS
416	c.34G>A (p.Gly12Ser)	*HRAS*	CS	Costello
417	c.1471 C>T (p.Pro491Ser)	*PTPN11*	NS	NS
418	c.1403 C>T (p.Thr468Met)	*PTPN11*	NS	NSML
419	c.922 A>G (p.Asn308Asp)	*PTPN11*	NS	NS
420	c.1649T>C (p.Leu550Pro)	*SOS1*	NS	NS
421	c.1403 C>T (p.Thr468Met)	*PTPN11*	NSML	NSML
422	c.508A>G (p.Lys170Glu)	*SOS1*	NS	NS
423	c.1492 C>T (p.Arg498Trp)	*PTPN11*	NS	NSML
424	c.922 A>G (p.Asn308Asp)	*PTPN11*	NS	NS
425	c.736G>C (p.Ala246Pro)	*BRAF*	NS	CFCS
426	c.1471C>A (p.Pro491Thr)	*PTPN11*	NS	NS
427	c.214A>C (p.Met72Leu)	*KRAS*	NS	NS
428	c.214 G>T (p.Ala72Ser)	*PTPN11*	NS	NS
429	c.1492 C>T (p.Arg498Trp)	*PTPN11*	NS	NS
430	c.188 A>G (p.Tyr63Cys)	*PTPN11*	NS	NS
431	c.1502A>G (p.Glu501Gly)	*BRAF*	NS	CFCS
432	c.1502A>G (p.Glu501Gly)	*BRAF*	NS	CFCS
433	c.184 T>G (p.Tyr62Asp)	*PTPN11*	NS	NS
434	c.417 G>C (p.Glu139Asp)	*PTPN11*	NS	NS
435	c.1294T>C (p.Trp432Arg)	*SOS1*	NS	NS
436	c.922 A>G (p.Asn308Asp)	*PTPN11*	NS	NS
437	c.836 A>G (p.Tyr279Cys)	*PTPN11*	NSML	NSML
438	c.1082G>C (p.Gly361Ala)	*RAF1*	NS	NS
439	c.786T>G (p.Asn262Lys)	*RAF1*	NS	NS
440	c.846 C>G (p.Ile282Met)	*PTPN11*	NS	NS
441	c.Glu76Asp (c.228 G>C)	*PTPN11*	NS	NS
442	c.228 G>T (p.Glu76Asp)	*PTPN11*	NS	NS
443	c.499_502delTGTT (p.Cys167GlnfsTer10)	*NF1*	NF	NF
444	c.922 A>G (p.Asn308Asp)	*PTPN11*	NS	NS
445	c.186_188del (p.Tyr63del)	*PTPN11*	NS	NS
446	c.1403C>T (p.Thr468Met)	*PTPN11*	NSML	NSML
447	c.1656 G>C (p.Arg552Ser)	*SOS1*	NS	NS
448	c.188 A>G (p.Tyr63Cys)	*PTPN11*	NS	NS
449	c.1510 A>G (p.Met504Val)	*PTPN11*	NS	NS
450	c.38G>A (p.Gly13Asp)	*NRAS*	NS	NS
451	c.806T>C (p.Met269Thr)	*SOS1*	NS	NS

a. Definitive diagnosis was established based on genetic results when they were unequivocal (e. g. p.GlySer in *HRAS*), on clinical reevaluation of patients, or both. However, updated clinical information was not available in all patients, and this label should be considered with caution as it could not be accurate in all cases.

NS: Noonan syndrome; CFCS: Cardiofaciocutaneous syndrome; CS: Costello syndrome; NSML: Noonan syndrome with multiple lentigines; NF: Neurofibomatosis type 1; NSLAH: Noonan syndrome-like with loose anagen hair; JMML: Juvenile myelomonocytic leukemia; NFNS: Neurofibromatosis-Noonan

síndrome. CFCS among others: generic suspicion of RASopathy, including CFCS; CS among others: generic suspicion of RASopathy, including CS; CFCS&CS among others: generic suspicion of RASopathy, including CFCS and CS.
